# Prescription Support Practice for Pharmacy Students: Pre-Post Educational Intervention Study

**DOI:** 10.2196/79545

**Published:** 2026-03-02

**Authors:** Fuka Aizawa, Kenta Yagi, Tsukasa Higashionna, Hirofumi Hamano, Shimon Takahashi, Yoshito Zamami, Kazuaki Shinomiya, Takahiro Niimura, Mitsuhiro Goda, Kei Kawada, Keisuke Ishizawa

**Affiliations:** 1 Clinical Research Center for Developmental Therapeutics, Tokushima University Hospital, Tokushima, Japan; 2 Department of Clinical Pharmacology and Therapeutics, Graduate School of Biomedical Sciences, Tokushima University, 3-18-15 Kuramoto-cho, Tokushima, 770-8503, Japan, +81-88-633-7061; 3 Department of Pharmacy, Shimane University Hospital, Shimane, Japan; 4 Department of Pharmacy, Okayama University Hospital, Okayama, Japan; 5 Department of Pharmacy, Tokushima University Hospital, Tokushima, Japan; 6 Department of Pharmaceutical Care and Clinical Pharmacy, Tokushima Bunri University, Tokushima, Japan; 7 Department of Clinical Pharmacology and Therapeutics, Graduate School of Biomedical and Heath Sciences, Hiroshima University, Hiroshima, Japan

**Keywords:** academic detailing, pharmaceutical clinical practice, prescription support, professional education, Interprofessional care

## Abstract

**Background:**

In the field of team-based care, pharmacists are vital for optimizing medication therapy. However, many medical professionals lack the opportunity to learn how to propose prescription changes with precision.

**Objective:**

This study aimed to address this knowledge gap by developing and assessing a new educational program for pharmacy students focused on prescription support and interprofessional collaboration.

**Methods:**

We recruited 191 fifth-year pharmaceutical students during the 2022‐2024 academic years. The program featured a 7-day intensive curriculum that included learning how to assist with prescriptions, analyzing clinical data, and engaging in role-playing exercises. A web-based questionnaire and a paper test were used to evaluate students’ awareness and knowledge both before and after the program. Statistical analyses were performed to verify the significance of changes; we utilized the Wilcoxon signed-rank test for the ordinal data derived from the specific behavioral objectives and 2-tailed paired *t* tests for the interval data from the knowledge tests. The magnitude of change was quantified using *r* for Wilcoxon tests and Cohen dz for 2-tailed *t* tests, with 95% CI calculated to ensure the stability and reliability of the observed results.

**Results:**

Analysis of the primary outcome specific behavioral objectives revealed statistically significant effects across all items (Wilcoxon signed-rank test; *P*<.001). Effect sizes (*r*=0.505‐0.835) ranged from moderate to large, with particularly large effects observed in identifying contents issue (*r*=0.835, 95% CI 0.126-0.330; *P*<.001). Knowledge test scores showed significant improvement in the following 3 subjects: pharmacology (*r*=−0.504, 95% CI –0.215 to 0.127; *P*<.001), organic chemistry (*r*=0.254, 95% CI –0.148 to –0.193; *P*=.004), and communication (*r*=0.221, 95% CI –0.151 to –0.190; *P*=.01). No significant changes were observed in pathology or pharmacokinetics.

**Conclusions:**

This program provides strong evidence that practical, hands-on learning with hospital pharmacists helps improve pharmacy students’ professional skills and optimize pharmaceutical therapies in interprofessional care. By teaching pharmacists to effectively propose prescription changes, the program equips them to become integral members of interprofessional care, ultimately leading to optimized pharmaceutical care for patients.

## Introduction

Collaborative care, provided by a team of medical professionals, can enhance therapeutic effects [1,2]. Interprofessional care—in which each medical professional contributes their expertise—optimizes medical services that are becoming more specialized and advanced. However, multidisciplinary cooperation requires an understanding of the role of each medical professional while maintaining individual expertise and using it effectively to provide better medical care. In interprofessional care, pharmacists are specialists in medicine. To optimize physicians’ prescriptions, pharmacists use their knowledge and skills—in dispensing medicines, formulating products, and managing supply—based on drug information [3-6]. Pharmacists can offer suggestions to ensure patient safety by promoting appropriate drug use and reducing adverse drug reactions [7]. Physicians view pharmacists’ abilities positively in terms of patient outcomes and time spent in practice [8]. The decrease in side effects and improvement in pharmacotherapy owing to pharmacist-prescribed interventions are evident [9,10]. Additionally, improving the expertise of pharmacists can enable physicians to pay greater attention to patient care and appropriate pharmacotherapy. Furthermore, pharmacists can support other medical professionals, improving health care economics by optimizing prescriptions and decreasing medicine-related incidents.

Academic detailing is a strategy for collaboration and learning between physicians and pharmacists. It promotes evidence-based prescribing by physicians, with pharmacists serving as academic detailers who provide physicians with information on drug evidence. Initiatives in various countries cover a wide range of areas, including antibiotics and opioids [11-13]. These approaches by pharmacists have been shown to significantly influence physicians’ prescribing behavior [14]. In Japan, pharmacists’ duties have shifted in recent years from dispensing medication and other object-oriented tasks to patient-centered activities, such as medication counseling. Pharmacists are required to be part of specialized teams, such as antimicrobial stewardship teams and nutrition support teams, underscoring their input as members of the health care team. However, in Japan, while such activities as the pre-consultation clinical laboratory test and proxy prescription entry are practiced [15-17], implementation remains limited to a few facilities, with most practices involving postprescription verification tasks. Opportunities for physicians and pharmacists to collaborate on tasks within the clinical setting are scarce, indicating a significant barrier to routine.

During their college years, many medical staff undertake practical clinical training after obtaining basic knowledge and training in simulated patient care [18-20]. Practical training during college gives students the opportunity to apply their knowledge and practical experience in a clinical situation, enhance their expertise, learn a good bedside manner, and consider their life values. This experience enables students to provide appropriate care when they begin working in the field as medical professionals and further develop their skills within the context of the rules and regulations of the institution to which they are assigned. Many countries require 4 to 6 years to educate pharmaceutical students. In Japan, a 6-year pharmaceutical education system was implemented in 2006 to strengthen clinical pharmacy skills. The curriculum integrates foundational sciences with a mandatory 22-week clinical clerkship in hospitals and community pharmacies during the fifth year. More professional programs are structured in the United States and the United Kingdom, where pharmacist-led activities are more advanced. However, in many other countries, while professional learning is planned, the lack of advanced or practical programs and professional collaboration is perceived as a problem [21]. Pharmacists have comprehensive medical and pharmacological knowledge, which can be enhanced by specialized training. However, learning about interprofessional care and individually optimized treatment is not part of the standard training program. It is difficult for beginners to optimize their professional skills in busy clinical environments and use them to improve patient health care safety. In fact, concerns have been raised in some countries about the lack of awareness of the pharmaceutical profession and the continuing professional development for pharmacists. The systematic development of pharmacists’ expertise and skills is necessary for them to become experienced and aware of processes when they begin clinical work. This clinical training focuses on reviewing the basic knowledge of pharmacists’ procedures in community pharmacies and hospitals and practicing how to manage patients. Students work with tutors to formulate drug therapy plans by monitoring the treatment of patients in their charge, providing information on the occurrence of adverse drug reactions, and proposing an appropriate drug therapy plan for patients from a variety of drugs. Pharmaceutical risk-benefit, dosage, form, and cost-effectiveness must be considered when providing prescription support. It is also important to fully understand the physician’s treatment plan and take steps to reflect the patient’s wishes, contextual factors, and financial situation as well as the latest evidence. Students should be able to understand these complex steps in a limited period and consider what action they could take as pharmacists to proactively intervene in prescription design; however, there is no well-developed training program that focuses on these issues. In addition, pharmacists do not have learning-enabling programs, and the effective transfer of knowledge and skills is not taking place.

This study was designed to investigate the impact of an integrated learning module on the prescription recommendation process within the basic clinical practice curriculum in Japan. The specific aims were to determine whether this intervention would enhance students’ awareness of interprofessional care and their knowledge and skills in pharmacotherapy planning. The outcome of the intervention was assessed through a pre- and postprogram evaluation using 2 questionnaires and a knowledge-based examination.

## Methods

### Study Design and Participants

This open-label, survey study was conducted at Tokushima University Hospital. In total, 191 pharmacy students (fifth-year students) who participated in clinical practice at Tokushima University Hospital in the 2022‐2023 academic year were enrolled in the program. The period of the study was a time of concern due to the coronavirus disease 2019 pandemic; however, none of the in-hospital regulations were in place, and all the procedures were conducted face-to-face. The students who desired clinical training at Tokushima University Hospital were recruited from 7 universities in Japan. They received 11 weeks of training in basic skills and patient care in the pharmacy before starting hospital training, in accordance with the training regulations. They were informed face-to-face about the study and given letters that obtained their consent to participate before the start of the practical training at the hospital. They were informed that all students would receive training as part of their clinical practice regardless of their participation in the study. Furthermore, refusal to provide consent would not impact their student performance in the clinical practice. Participants who provided informed consent were included in the analysis. We excluded those who did not provide informed consent, with missing data in the process of data collection, or whose lack of attendance made before-and-after comparisons impossible. All facilitators leading this program were pharmacists with doctoral degrees and at least 5 years of clinical experience. Furthermore, the lead facilitator held certification from the Japan Academic Detailing Research Association. This study defined a 5-point improvement in test scores as the effect size for the educational program. With an SD of 12 points, 80% power, and an α error of .05, the sample size was calculated. The study required 91 participants. Anticipating approximately 6 dropouts per year, the plan was to recruit 109 participants over 3 years (Checklist 1).

### Procedures for Academic Detailing Program

All students joined an “academic detailing program” to learn the process of proposing prescriptions to physicians together with clinical practice in accordance with the guidelines for practical training of the Council on Pharmaceutical Education in Japan during the 11-week clinical practice period. No control group was established to ensure equal opportunities for learning within the same environment. The face-to-face program was designed to promote deeper thinking and increased cooperation with others. The students were randomly divided into small groups of 5 to 6, depending on the number of students accepted during the training period. Grouping involved researchers assigning numbers to participants and randomizing them to include both male and female participants. The program comprised three lectures over 5 days: (1) 2 days of face-to-face lectures and practice on how to review clinical papers and apply them to patients (1 pharmacist on staff); (2) half a day of face-to-face lectures on pharmacology, pathogenesis, chemistry, pharmacokinetics, and communication (30 minutes each, 4 pharmacists on staff); (3) 1 day of obtaining information and discussing the recommended drugs; and (4) preparation of materials for prescription proposals to physicians (or a staff pharmacist) and role-playing of prescription proposals among students. Two types of papers that had been accepted for submission were used to review the clinical papers [22,23]. During the paper review, the pharmacist gave lectures on reading papers, basic knowledge (diseases and drugs), guidelines, and other clinical studies related to the topic of the review. Each group read the papers and summarized the information on the worksheet. Then, the students discussed whether the evidence should be applied to the patient, including factors such as cost-effectiveness and patient willingness. The prescription proposal process focused on constipation. Students collected information on drugs used in Japan (excluding herbal medicines), including chemical structure, adaptation, dosage, administration, and contraindications. The students assessed the patients, discussed the drug therapy plans, and prepared handouts to assist physicians in prescribing the drugs. Based on the prescription proposal documents prepared by the program, students and pharmacists acting as physicians proposed prescriptions for 10 to 15 minutes and discussed therapeutic planning. After the session, the physicians decided whether or not to prescribe the prescriptions. Finally, the pharmacist who created the program provided feedback on improvements to the students’ prescribing suggestions and shared examples of actual clinical experience.

### Outcomes

The primary outcome measure was a change in specific behavioral objectives (SBOs) required in interprofessional care and prescribing support before and after clinical practice, with students requiring a score of at least 3 before undertaking the education program. The SBO change was collected using a web response, using a 5-point scale ranging from 1 (not possible) to 5 (possible). The questionnaire was developed by hospital pharmacists and was not related to teaching. It was based on common SBOs for pharmacy education, which were chosen from the Ministry of Education, Culture, Sports, Science and Technology, Japan [17]. We specifically selected SBOs that directly pertain to pharmacotherapy planning and prescription recommendation skills. This prioritization is based on the consensus that these competencies are the core clinical responsibilities of pharmacists in modern team–based medicine. By focusing on these high-priority items, we aimed to evaluate the practical and clinical readiness of students in scenarios that most closely mirror real-world pharmaceutical care. We selected 37 items related to our program from parts 1 to 10 (Table S1 in Multimedia Appendix 1). The original SBOs (Table S2 in Multimedia Appendix 1) comprised 11 items from part 11. The knowledge test included 30 questions, consisting of 6 questions each on chemistry, pharmacokinetics, pharmacology, pathology, and communication. To assess knowledge proficiency, 3 of the 6 questions were related to content not handled in the program but studied at university and in the clinical setting. Questionnaires and tests were administered at the beginning and end of the clinical practice. The names of the students were written on the questionnaires and tests at the time of collection for pre-post comparisons but were given serial numbers for anonymization in the analysis.

### Ethical Considerations

This study was conducted in compliance with the Ethical Guidelines for Medical Research Involving Human Subjects and with the approval of the Ethics Committee of Tokushima University Hospital (approval number: 4193, certificate date: June 27, 2022). Informed consent was obtained from all individual participants included in the study. The authors affirm that human research participants provided informed consent for publication of the images and all results. All data obtained and analyzed in the present study are included in this manuscript and not published elsewhere. All data were **de-identified** to ensure participant privacy. Personally identifiable information was replaced with **unique alphanumeric codes** (pseudonymization) to ensure that individuals cannot be identified from the dataset. Access to the key-code list was strictly restricted to the primary investigators. **No compensation**, monetary or otherwise, was provided to the participants for their involvement in this human subjects research. Participation was entirely voluntary.

### Statistical Analysis

The primary outcome measure, the SBO questionnaire, is a 5-point ordinal scale. We performed a Wilcoxon signed-rank test for paired comparisons without assuming a specific distribution. The secondary outcome measure, the knowledge test, is an interval scale based on the number of correct answers. As the total score is out of 30 points, assuming normality, we used a 2-tailed paired *t* test. The subject-specific total scores are out of 6 points. Given the low number of scale levels and the high likelihood that normality assumptions would not hold, we analyzed these scores using the Wilcoxon signed-rank test. Similarly, the subject-specific total scores divided into related or unrelated categories are also out of 3 points; thus, we analyzed these scores using the Wilcoxon signed-rank test. The purpose of this study is to conduct an exploratory evaluation of the impact of the developed educational program. As this research design is not intended to verify intervention effects, adjustments for multiple comparisons were not necessary. For the pre-post difference in total knowledge test scores (out of 30 points), histograms and Q-Q plots were created to visually confirm normality. For all evaluation items, the standardized effect size and its 95% CI were calculated following the procedure set out below. For the SBO questionnaire using the Wilcoxon signed-rank test, the test’s subject-specific total scores, and the related or unrelated subject-specific total scores, the effect size *r*=*Z*/sqrt(N) and its 95% CI were calculated. Here, *Z* is the standardized value of the Wilcoxon signed-rank test statistic (standardized test statistic), and N is the number of participants. The 95% CI was constructed using the normal approximation. For the total scores on the knowledge test, which used a 2-tailed paired *t* test, we calculated the effect size Cohen dz=*t*/sqrt(N) and its 95% CI. Here, *t* is the paired *t* test statistic, and N is the number of participants. The 95% CI was constructed using the normal approximation. The statistical software IBM SPSS Statistics (version 28.0.1.0; IBM Corp.) was used for the analysis.

## Results

### Participants’ Characteristics

**Figure 1.**
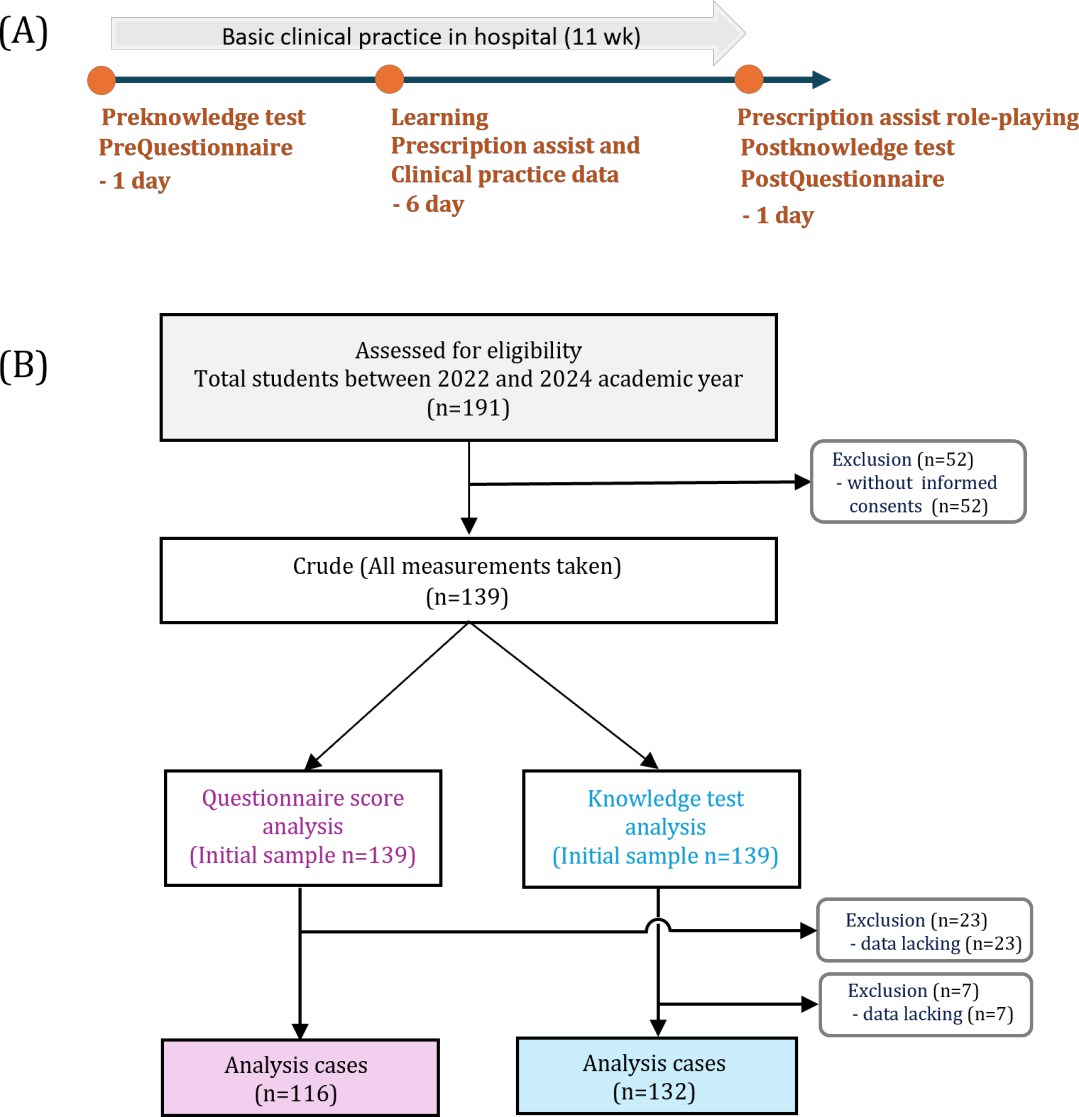
(A) Study design and (B) participant selection.

The study was conducted from May 23, 2022, to February 11, 2024, and 191 students were included (Figure 1). As 52 participants did not provide consent to participate, they were excluded, resulting in 139 participants (Table S2 in Multimedia Appendix 1). The questionnaire and tests were administered to all students during clinical practice. Approximately 116 cases (58 male participants, 50%) were included in the questionnaire analysis, after excluding 23 cases (6 male participants, 26.1%) with invalid responses before or after the intervention. For the test analysis, 132 cases (59 male participants, 44.7%) were included, excluding 7 cases (4 male participants, 57.2%) with no data before or after the intervention. Among the data used for analysis, there were 87 (75%) affiliated students for the questionnaire and 100 (75.7%) for the knowledge test (Table S3 in Multimedia Appendix 1).

### Raising Students’ Awareness as Medical Professionals

For participants with baseline scores but no postintervention scores, the missing postintervention scores were imputed using the baseline scores (baseline observation carried forward), and the analysis was performed. The results showed no significant deviation from the original analysis. To evaluate the reliability of the measurement tools, Cronbach α coefficients were calculated. The SBO questionnaire demonstrated good-to-excellent internal consistency, with alpha values ranging from 0.700 to 0.912 across its factors.

**Figure 2.**
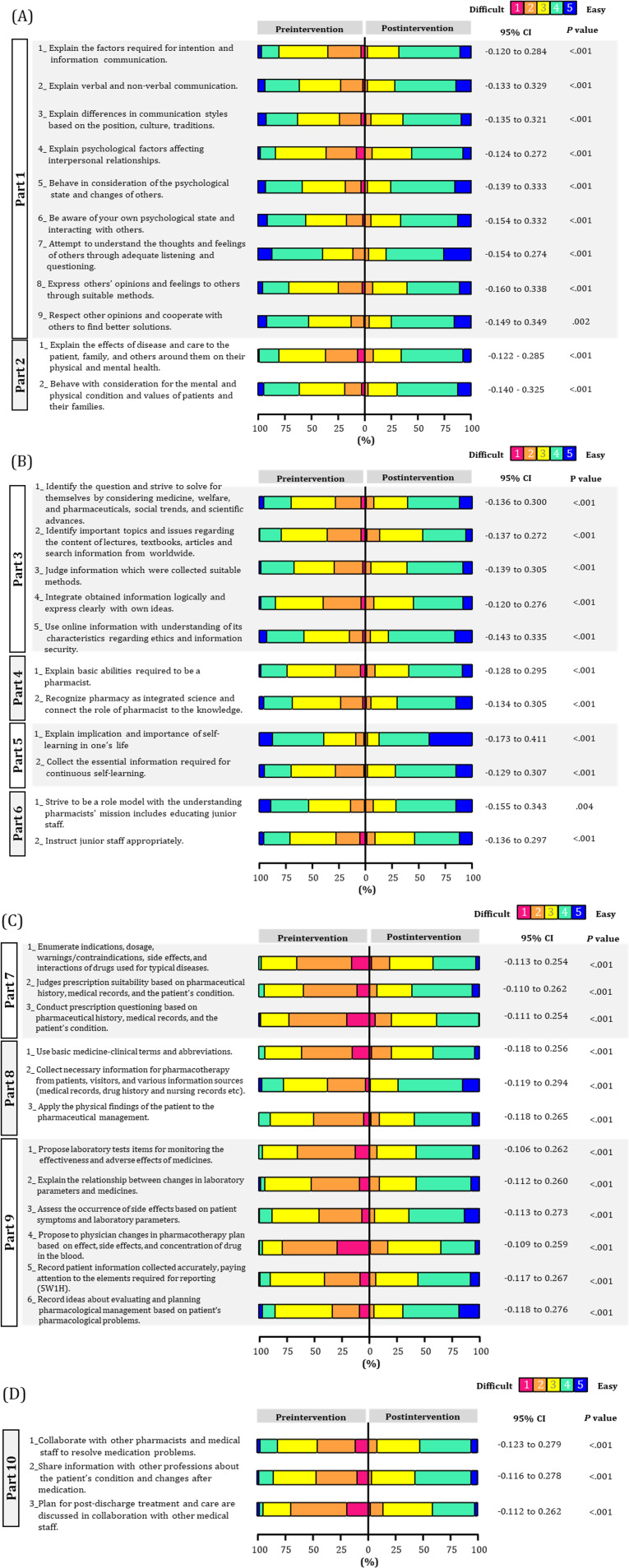
Changes in specific behavioral objectives in pharmacy students. Each color bar shows the changes in self-evaluation in (A) building trust with others, (B) active learning and development of future leaders, (C) handling medical information and planning drug therapy, and (D) interprofessional care. On a 5-point scale, the lower number indicates “difficult.” The x-axis shows the percentage of each respondent when the total is set at 100% (n=116).

The analysis of the primary outcome revealed statistically significant effects across all items (Q1-1 to Q11-11; Figures2  3, Multimedia Appendix 1; all *P*<.001). Effect sizes ranged from moderate to large (effect size: 0.505‐0.835), with particularly large effects observed in Q3-2 (effect size: 0.835, 95% CI 0.126-0.330). The lower bound of the 95% CI exceeded zero for all items, suggesting that the observed effects were not due to chance but rather stable effects. For the secondary outcomes, unlike the primary outcome, no statistically significant effects were observed for most items (eg, Q2-1; *P*=.59, Table S1 in Multimedia Appendix 1). Effect sizes were widely distributed across both positive and negative values (effect size: –0.746 to 1.000), and the 95% CI for items showing no significant difference mostly crossed zero. Statistically significant effects were observed only for Q1-3 (effect size: 0.332, 95% CI 0.055-0.609; *P*=.02).

**Figure 3.**
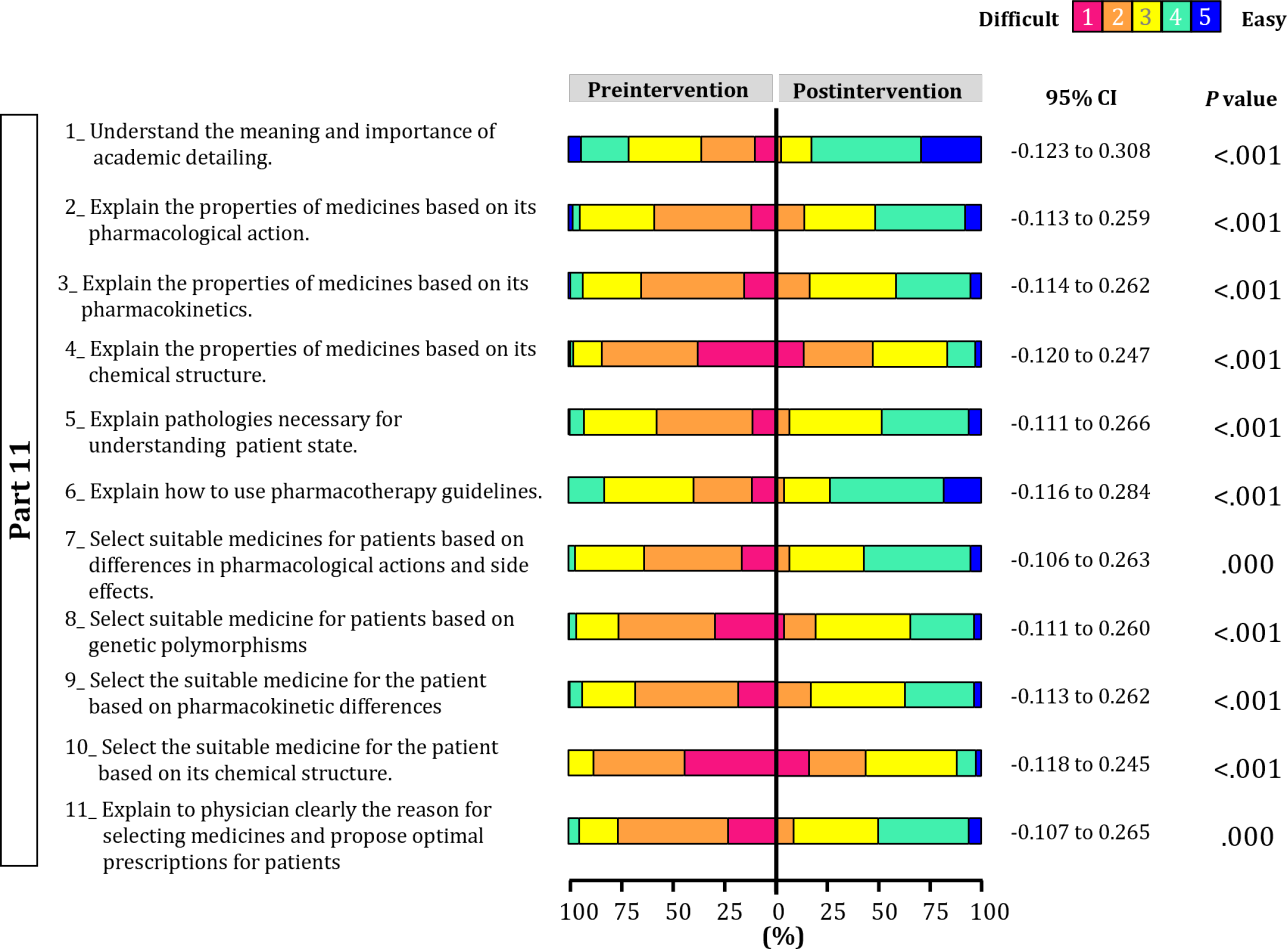
Changes in original specific behavioral objectives in pharmacy students.

### Knowledge Building for Prescribing Planning in Students

**Figure 4.**
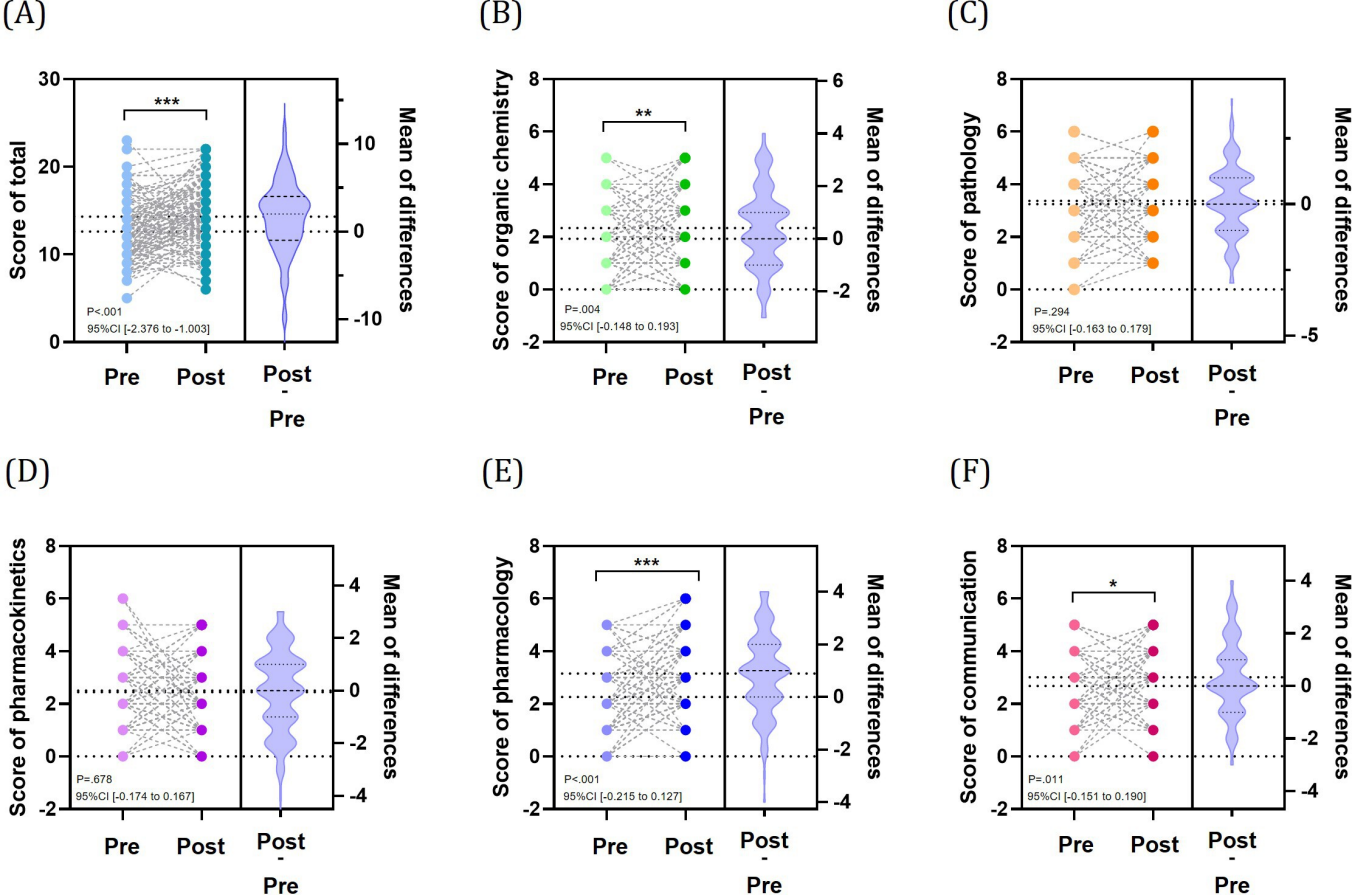
Alteration of knowledge proficiency in basic pharmacy. (A) Total score in basic pharmacy using a paired *t* test. Score of each domain using a Wilcoxon signed-rank test: (B) organic chemistry, (C) pathology, (D) pharmacokinetics, (E) pharmacology, and (F) communication. Violin plots for each figure indicate the mean of score difference before and after intervention. *P<.05, **P<.01, ***P<.001.

**Figure 5.**
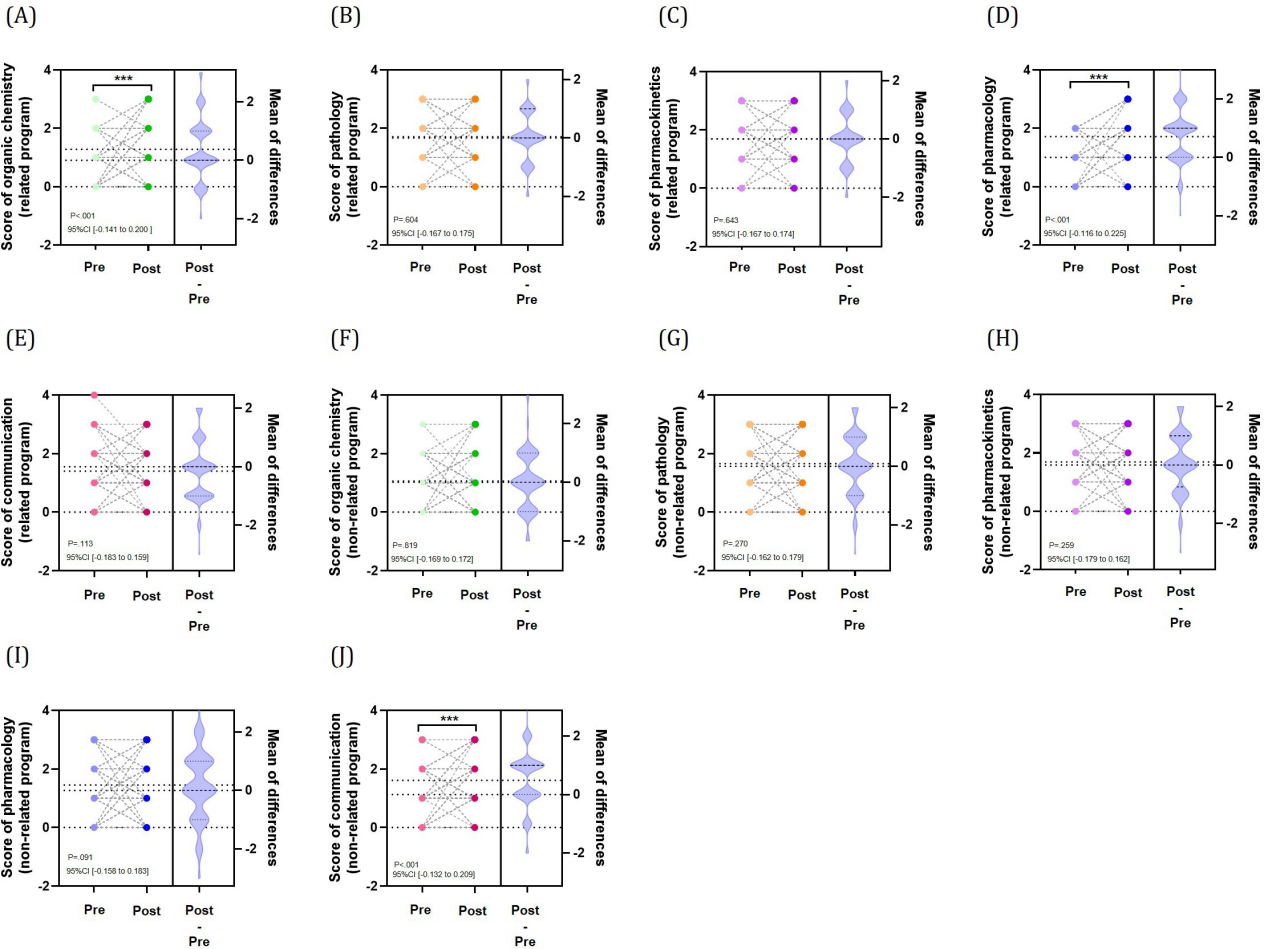
Knowledge proficiency analysis of subgroups in each domain (n=132). Changes in program-related knowledge proficiency questions: (A) organic chemistry, (B) pathology, (C) pharmacokinetics, (D) pharmacology, and (E) communication. Changes in program nonrelated knowledge proficiency questions: (F) organic chemistry, (G) pathology, (H) pharmacokinetics, (I) pharmacology, (J) and communication. Violin plots of each figure indicate the mean of score difference before and after intervention. A Wilcoxon signed-rank test was used for organic chemistry in changes in program-related knowledge proficiency questions and for pathology and communication in program nonrelated knowledge proficiency questions. ***P<.001.

In the knowledge test of prescription support in the related program, no significant deviation from a normal distribution was observed for the pre-post difference in total knowledge test scores (out of 30 points). The 30-item knowledge test showed a Cronbach α of .427. Knowledge test scores (Figure 4) showed significant improvement in the following 3 subjects: pharmacology (effect size: −0.504, 95% CI −0.215 to −0.127; *P*<.001), organic chemistry (effect size: 0.254, 95% CI −0.148 to −0.193; *P*=.004), and communication (effect size: 0.221, 95% CI −0.151 to −0.190; *P*=.01). Meanwhile, no significant effects were observed for pathology (*P*=.29) and pharmacokinetics (*P*=.68). Program-specific analysis (Figure 5) revealed extremely significant effects: organic chemistry in the related program (effect size: 0.342; *P*<.001) and communication in the nonrelated program (effect size: 0.446; *P*<.001), with clear differences in effect size observed between programs. Particularly in pharmacology, while the overall results showed a large negative effect, the related program demonstrated a highly significant large positive effect (effect size: 0.624; *P*<.001), exhibiting the most pronounced difference between programs (Table S3 in Multimedia Appendix 1).

## Discussion

### Principal Findings

This study aimed to evaluate a specialized educational program designed to enhance pharmacy students’ ability to apply professional knowledge in interprofessional care settings during hospital practice. Our findings indicate that the program significantly improved students’ self-reported confidence and objective knowledge levels. Notably, while over 70% of the students initially felt their communication skills were insufficient, this perception shifted positively following the intervention. The objective knowledge tests mirrored this improvement, particularly in pharmacology and the ability to apply pharmaceutical information to drug therapy planning.

They had opportunities to share information correctly and concisely with medical staff and worked in groups to summarize and present their research, analogous to a medical team environment. While students could learn the necessary elements for prescription support, the collaboration skills were generic. The program included students and pharmacists, using a simulated prescription proposal. These skills are obtained in clinical practice, and it is difficult to obtain them with static learning at universities. The importance of clinical practice has been mentioned in various countries, and optimal educational methods are currently under consideration [24-26]. Students are aware that health care professionals require continuous learning and education but lack understanding of how to gather information. Hospital practice requires learning about management in commercial pharmacies, and this requires evidence. We provided students with clinical research papers, secondary sources, and various guidelines. The students examined the validity of the evidence and determined whether it was appropriate. This helped them discriminate between sources to use them effectively. Almost all students answered with an understanding and ability to apply the medical and pharmaceutical information to plan drug therapy. By contrast, improvements in the ability to inform and discuss with medical staff were less significant. The students participated in conferences on antibiotic stewardship and nutritional management to learn the role of pharmacists in collaborative care. We designed the program within a limited duration to complete the basic program specified by the educational institutions of Japan. However, the limited time for implementing multidisciplinary collaboration was insufficient and might have limited the status of improvement of practical items.

Clinical practice is expected to improve the knowledge of students with or without the present program. However, if pharmacists do not strongly intervene in student learning, then improvement in items not related to the program might not be expected. We assessed the improvement in students’ knowledge levels by administering a knowledge test on the content covered and not covered in the program to supplement the self-assessments. This confirmed whether the self-reported knowledge acquisition was correct. The test scores increased significantly after the intervention; however, there were no significant differences between the 2 domains in terms of pharmacokinetics and pathophysiology. Students were expected to already know about medicines, and we chose constipation as the theme, because it is a common ailment in children and adults, and fewer types of medicines are available. Most drugs used to treat constipation act on the colon, and there are few drug-drug interactions. This might have made it difficult for students to understand the characteristics of each drug. However, the average score distribution of pathology regarding both program-related and nonprogram-related content was higher than that of the other regions in the preintervention period. There were no marked differences between the pre- and postintervention values. Considering that the relevance of physiology and pathophysiology is important for understanding the pharmacological effects of drugs, the pharmacology scores were not high before the intervention. The average pharmacology score improved at the end of the program, particularly for program-related questions. This may be due to the greater focus on assessing drug interactions to recommend a suitable drug to the simulated patient when preparing prescription recommendations for the physician. Drug interactions include drug formulation characteristics, such as dosage form, pH, chemical modifications, induction or antagonism of metabolic enzymes, and overlap of pharmacological action targets. The practice of prescribing proposals for a simulated patient—based on their concomitant medications and therapies—requires a problem-solving approach to learning, rather than a classroom lecture. Although there was an improvement in the knowledge of content not covered in the program, it was not as great as that of the program-covered content. This suggests that learning about pharmacology is more meaningful when it is combined with the pathophysiology of a disease or drug formulation. The distribution of scores on the organic chemistry questions was sparser than that in the other areas, although there was some improvement after the intervention. The results of the questionnaire on understanding structure-based features or selection improved after the intervention; however, more than half the students’ answers in the questionnaire test were “below average” (mean score <3). The focus of organic chemistry at college is on reactions in the synthesis and properties of the synthesized compounds (eg, the presence of isomers, stability, and insolubility), not on understanding the structure, as it relates to pharmacology and kinetics. Drugs are synthesized to be stable and persistent by adding chemical modifications, such as insulin and statins, to endogenous molecules in the body. It is easy for students to speculate about the relationship between the structural properties and the disease or pharmacokinetics of the drug. However, the structures of natural molecules synthesized by molecular structural analysis are often complex, and it is difficult to link this knowledge to that in areas other than organic chemistry. Understanding the drug structure is necessary to estimate changes in formulation, drug stability, and pharmacokinetics. In this program, lectures on each specialty area were provided by hospital pharmacists, who discussed the structural characteristics of each laxative and the properties related to its structure in organic chemistry. The results of the preimplementation questionnaire revealed that the students lacked expertise in organic chemistry and pharmacokinetics. Lectures by hospital pharmacists may be useful for students to understand the meaning of organic chemistry in pharmacotherapy. However, this could be better remedied through combined lectures at universities—such as linking organic chemistry to pharmacology and pharmacokinetics—or collaborating with academic or clinical teachers in practice. Collaboration between universities and medical institutions is not well advanced. Sharing this program between both institutions might enable them to not only practice seamless clinical education but also to produce pharmacists with a research focus.

### Limitations

This study has some limitations. First, it evaluated the educational effects of adding practical content as advanced learning to a basic program. To avoid differences in education, the results of a single group were compared before and after intervention. We were required to provide instruction on all items mandated within the prescribed period by Japanese educational institutions. Consequently, we needed to ensure equal educational opportunities for all participants, making it difficult to use a control group for comparison. While this study demonstrates the impact of the educational program we developed, it cannot fully exclude the influence of the natural course of the training period or other confounding factors. Nevertheless, the program-related content scored better in the question rating whether the content was program-related or not in the knowledge test. It seems there was a direct improvement in the program implementation. This study should be considered a pilot test, necessitating the implementation of more rigorous clinical research in the future. The second limitation was the duration of students’ knowledge retention. The clinical practice was 11 weeks, and the evaluations were conducted at the beginning and end. Improvements in knowledge and understanding of the pharmacist’s role in interprofessional care were observed during this period. However, we were unable to track whether the students sustained their learning when they became medical professionals. Therefore, a nationwide system for continuous data collection after graduation is required. The third limitation is the collaboration with other medical professionals. The program was only conducted with pharmacy students and hospital pharmacists. Although pharmacy students may spend time at medical team conferences, it is necessary to establish a long-term cross-sectional committee to assemble the contents of a common learning program. Much of the reporting on multidisciplinary collaboration is based on medical schools [27]. A previous study reported on interprofessional education by medical and pharmacy students [28]. It is necessary to collaborate with institutional staff so as to establish this kind of learning system in the future. The fourth limitation is that this study concerns the internal consistency of the 30-item paper test. The calculated Cronbach α was .427, which is below the generally accepted threshold of .70. This low value may be attributed to the multidimensional nature of the test, which was designed to assess a broad range of knowledge rather than a single construct. Future studies should consider using validated standardized tests or refining the test items to improve reliability. Finally, this study evaluated the educational effects of the original program at a single center. The participating students belonged to different universities and a certain level of educational effect was expected for all students. However, it is unclear whether the same effect can be achieved at different hospitals because the medical institutions provided different educational resources. We plan to conduct this program at several medical institutions to determine its usefulness as an educational item for basic rather than advanced programs.

### Conclusions

The key contribution of this study is that it measured and documented significant improvements in the ability of pharmacy students to provide direct collaboration with the members of interprofessional care, particularly with respect to drug information and prescription consultations. Our findings provide evidence that practical, hands-on learning facilitated by hospital pharmacists rather than static university lectures is essential for optimizing pharmaceutical therapies. By equipping students with the skills to propose evidence-based prescriptions, this program prepares them to become integral members of the health care team. Furthermore, as physicians increasingly recognize that pharmacist-led prescription support can optimize clinical processes, reduce medical costs, and minimize side effects [29]. By integrating the specialized expertise of physicians and pharmacists, this program represents an important step toward a seamless, coordinated, and advanced medical care system. Ultimately, fostering these collaborative competencies in clinical practice will lead to more individually optimized pharmacotherapy and improved patient outcomes in the evolving landscape of global interprofessional care.

## Supplementary material

10.2196/79545 Multimedia Appendix 1 Supplementary tables on (1) additional information of specific behavioral objectives (2) additional information of specific behavioral objectives to prescription support practice, (2) basic characteristics of pharmacy students, and (4) all cases of analysis.

10.2196/79545 Checklist 1 CONSORT checklist.

## Abbreviations

SBO: specific behavioral objective
