# Supplementary material for: Prescription Support Practice for Pharmacy Students: Pre-Post Educational Intervention Study
Source: JMIR Med Educ. 2026 Mar 2;12:e79545. doi: 10.2196/79545 (PMC12954723; doi:10.2196/79545)
Supplement: Multimedia Appendix 1 [file mededu-v12-e79545-s001.docx]

Table S1. *Additional information of Specific Behavioral Objectives*

| Basics | | | |  |
| --- | --- | --- | --- | --- |
| Part 3. Establishing a trust relationship | Knowledge | Skill | Attitude | Questionnaire  No |
| [*Communication*] | | | |  |
| Explain the factors required for intention and information communication. |  |  |  | Part1-1 |
| Explain verbal and non-verbal communication. |  |  |  | Part1-2 |
| Explain differences in communication styles based on the position, culture, traditions. |  |  |  | Part1-3 |
| Explain psychological factors affecting interpersonal relationships. |  |  |  | Part1-4 |
| Behave in consideration of the psychological state and changes of others. |  |  | ✓ | Part1-5 |
| Be aware of your own psychological state and interacting with others. |  |  | ✓ | Part1-6 |
| Attempt to understand the thoughts and feelings of others through adequate listening and questioning. |  | ✓ | ✓ | Part1-7 |
| Express one's opinions and feelings to others through suitable methods. |  | ✓ | ✓ | Part1-8 |
| Respect others’ opinions and cooperate with others to find better solutions. | ✓ | ✓ | ✓ | Part1-9 |
| [*Patients, consumers, and pharmacists*] | | | |  |
| Explain the effects of disease and care to the patient, family, and others around them on their physical and mental health. |  |  |  | Part2-1 |
| Behave with consideration for the mental and physical condition and values of patients and their families. |  | ✓ |  | Part2-2 |
| Part 5. Active learning and development of human resources | | | | |
| [*Learning styles*] | | | |  |
| Identify the question and strive to solve for themselves by considering medicine, welfare, pharmaceuticals, social trends, and scientific advances. |  |  | ✓ | Part3-1 |
| Identify important topics and issues regarding the content of lectures, textbooks, articles, and search for information worldwide. |  | ✓ |  | Part3-2 |
| Judge information that was collected through suitable methods. | ✓ | ✓ |  | Part3-3 |
| Integrate obtained information logically and express clearly with own ideas. |  | ✓ |  | Part3-4 |
| Use online information with an understanding of its characteristics regarding ethics and information security. | ✓ |  | ✓ | Part3-5 |
| [*Overview of pharmacy education*] | | | |  |
| Explain the basic abilities required to be a pharmacist. |  |  |  | Part4-1 |
| Recognize pharmacy as an integrated science and connect the role of the pharmacist to the knowledge. | ✓ |  | ✓ | Part4-2 |
| [*Active learning*] | | | | |
| Explain the implications and importance of self-learning in one's life |  |  |  | Part5-1 |
| Collect the essential information required for continuous self-learning. |  | ✓ |  | Part5-2 |
| [*Development of human resources*] | | | | |
| Strive to be a role model by understanding pharmacists’ mission, which includes educating junior staff. |  |  | ✓ | Part6-1 |
| Instruct junior staff appropriately. |  | ✓ | ✓ | Part6-2 |
| Clinical pharmacy | | | | |
| Part2. Preparation of medicines on prescription | | | | |
| [*Prescriptions and questionnaires*] | | | | |
| Enumerate indications, dosage, warnings/contraindications, side effects, and interactions of drugs used for typical diseases. |  |  |  | Part7-1 |
| Judge prescription suitability based on pharmaceutical history, medical records, and the patient’s condition. | ✓ | ✓ |  | Part7-2 |
| Conduct prescription questioning based on pharmaceutical history, medical records, and the patient’s condition. |  | ✓ | ✓ | Part7-3 |
| Part 3. Pharmacotherapy practice | | | | |
| [*Understanding patients’ information*] | | | | |
| Use basic medicine-clinical terms and abbreviations. | ✓ |  | ✓ | Part8-1 |
| Collect necessary information for pharmacotherapy from patients, visitors, and various information sources (medical records, drug history, and nursing records). |  | ✓ | ✓ | Part8-2 |
| Apply the physical findings of the patient to the pharmaceutical management. |  | ✓ | ✓ | Part8-3 |
| [*Prescribing planning and pharmacotherapy practice (evaluation of efficacy and side effects)*] | | | |  |
| Propose laboratory test items for monitoring the effectiveness and adverse effects of medicines. | ✓ |  | ✓ | Part9-1 |
| Explain the relationship between changes in laboratory parameters and medicines. |  |  |  | Part9-2 |
| Assess the occurrence of side effects based on patient symptoms and laboratory parameters. |  |  |  | Part9-3 |
| Propose to physician changes in pharmacotherapy plan based on effect, side effects, and drug concentration in the blood. | ✓ |  | ✓ | Part9-4 |
| Record patient information collected accurately, paying attention to the elements required for reporting (5W1H). |  | ✓ |  | Part9-5 |
| Record ideas about evaluating and planning pharmacological management based on patient's pharmacological problems. | ✓ | ✓ |  | Part9-6 |
| Part 4. Participation in team care | | | | |
| [*Team care in medical institutions*] | | | | |
| Collaborate with other pharmacists and medical staff to resolve medication problems. |  |  | ✓ | Part10-1 |
| Share information with other professions about the patient’s condition and changes after medication. | ✓ |  | ✓ | Part10-2 |
| Plan for post-discharge treatment and care are discussed in collaboration with other medical staff. | ✓ |  | ✓ | Part10-3 |

Table S2. *Additional information of Specific Behavioral Objectives to prescription support practice*

| Prescription support | | | | |
| --- | --- | --- | --- | --- |
| [*Implication of academic detailing*] | Knowledge | Skill | Attitude | Questionnaire  No |
| Understand the meaning and importance of academic detailing. | ✓ |  | ✓ | Part11-1 |
| [*Characterization of medicines and basic pharmacology*] | | | | |
| Explain the properties of medicines based on their pharmacological action. | ✓ |  |  | Part11-2 |
| Explain the properties of medicines based on their pharmacokinetics. | ✓ |  |  | Part11-3 |
| Explain the properties of medicines based on their chemical structure. | ✓ |  |  | Part11-4 |
| [*Characterization of medicines and patients’ information*] | | | | |
| Explain pathologies necessary for understanding patient state. | ✓ |  |  | Part11-5 |
| Explain how to use pharmacotherapy guidelines. | ✓ |  |  | Part11-6 |
| Select suitable medicines for patients based on differences in pharmacological actions and side effects. | ✓ | ✓ |  | Part11-7 |
| Select suitable medicine for patients based on genetic polymorphisms | ✓ | ✓ |  | Part11-8 |
| Select the suitable medicine for the patient based on pharmacokinetic differences | ✓ | ✓ |  | Part11-9 |
| Select the suitable medicine for the patient based on its chemical structure. | ✓ | ✓ |  | Part11-10 |
| [*Practice of prescription support*] | | | |  |
| Clearly explain to physician the reason for selecting medicines and propose optimal prescriptions for patients. | ✓ | ✓ | ✓ | Part11-11 |

Table S3_Basical Characteristic of pharmacy students

|  | Questionnaire score analysis | | Knowledge test analysis | |
| --- | --- | --- | --- | --- |
|  | (n=139) | | (n=139) | |
|  | Analysis case | Excluded case | Analysis case | Excluded case |
|  | 116 | 23 | 132 | 7 |
| Sex, male (%) | 58 (50.0) | 6 (26.1) | 59 (44.7) | 4 (57.2) |
| Affiliation |  |  |  |  |
| - university-affiliated (%) | **87 (75.0)** | **16 (70.0)** | **100 (75.7)** | **3 (42.8)** |
| 2022 | 28 | 7 | 33 | 1 |
| 2023 | 25 | 6 | 32 | 0 |
| 2024 | 34 | 3 | 36 | 2 |
| -university-non-affiliated | **29 (25.0)** | **7 (30.0)** | **31 (24.3)** | **4 (57.2)** |
| 2022 | 9 | 5 | 11 | 3 |
| 2023 | 12 | 0 | 12 | 0 |
| 2024 | 8 | 2 | 8 | 1 |

Table S4_All cases of analysis

| Analysis subject | Total cases (n) | Exclusion cases (n) | Analysis cases (n) |
| --- | --- | --- | --- |
| Questionnaire score | 139 | 23 | 116 |
| Knowledge test | 139 | 7 | 132 |
